# Early Drowsiness Detection via Second-Order Derivative Analysis of Heart Rate Variability: A Non-Contact ECG Approach with Machine Learning

**DOI:** 10.3390/s26041348

**Published:** 2026-02-20

**Authors:** Fabrice Vaussenat, Abhiroop Bhattacharya, Julie Payette, Alireza Saidi, Victor Bellemin, Geordi-Gabriel Renaud-Dumoulin, Sylvain G. Cloutier, Ghyslain Gagnon

**Affiliations:** 1 Department of Electrical Engineering, École de Technologie supéRieure, Montreal, QC H3C 1K3, Canadaghyslain.gagnon@etsmtl.ca (G.G.); 2 IRSST (Institut de Recherche Robert-Sauvé en Santé et en séCurité du Travail), 505 Blvd. De Maisonneuve Ouest, Montreal, QC H3A 3C2, Canada; alireza.saidi@irsst.qc.ca

**Keywords:** drowsy driving detection, heart rate variability, HRV derivatives, non-contact ECG, capacitive sensing, machine learning, driver monitoring systems, road safety

## Abstract

Drowsy driving contributes to roughly 20% of traffic fatalities, yet most detection systems rely on behavioral cues that appear only after impairment has set in. Here we ask whether first and second derivatives of heart rate variability (HRV) can detect pre-crash states earlier than conventional approaches. Twenty-five participants completed 49 driving simulator sessions while we recorded cardiac activity through capacitive ECG electrodes embedded in the seat backrest—a non-contact method that avoids the privacy concerns of camera-based monitoring. To prevent circular evaluation, ground truth labels were based solely on crash proximity rather than HRV-derived scores. The combined HRV feature set (conventional metrics plus derivatives) achieved AUC = 0.863 for pre-crash prediction; derivatives alone reached only AUC = 0.573, indicating their value as complementary rather than standalone features. Driving performance indicators remained the strongest predictors (AUC = 0.999). Temporally, derivative-based detection preceded behavioral manifestations by 5–8 min and crash events by 6.8 ± 2.3 min. Across 1591 crashes and 6.78 million data points, we found that HRV derivatives capture physiological changes that precede overt impairment, though their utility depends on integration with other feature types.

## 1. Introduction

Drowsy driving represents one of the most significant yet underaddressed threats to road safety worldwide. The National Highway Traffic Safety Administration (NHTSA) estimates that drowsy driving causes approximately 100,000 crashes, 71,000 injuries, and 1550 fatalities annually in the United States alone, with actual numbers likely higher due to underreporting [1]. European studies report similar patterns, with fatigue implicated in 15–20% of all traffic accidents and up to 25% of fatal highway crashes [2,3,4]. The economic burden exceeds $109 billion annually in the US, encompassing medical costs, property damage, lost productivity, and quality of life impacts [5].

Regulatory bodies have responded to these statistics with mandatory Driver Monitoring System (DMS) requirements. The European Union’s General Safety Regulation (GSR), effective July 2024, mandates Driver Drowsiness and Attention Warning (DDAW) systems in all new vehicle types, with full fleet compliance required by July 2026 [6,7]. These systems must warn drivers when drowsiness reaches Level 8 or higher on the Karolinska Sleepiness Scale (KSS) and activate automatically above 70 km/h. The EU expects these measures to prevent over 25,000 fatalities and 140,000 serious injuries by 2038. Similar regulations are emerging in China (GB/T 26773-2024), Japan, and through Euro NCAP protocols that require direct DMS for five-star safety ratings [8]. This regulatory momentum has accelerated research into robust, privacy-preserving drowsiness detection technologies.

Unlike alcohol or drug impairment, drowsiness develops gradually and often without driver awareness. Studies demonstrate that moderately drowsy drivers significantly underestimate their impairment level, with subjective sleepiness ratings showing weak correlation with objective performance decrements [9]. This perceptual gap creates a critical safety challenge: drivers frequently fail to recognize dangerous drowsiness levels until performance is already severely compromised.

Current commercial Driver Monitoring Systems (DMS) predominantly rely on camera-based behavioral analysis, detecting drowsiness through indicators such as PERCLOS (percentage of eye closure), yawning frequency, head pose, and blink patterns [10,11,12]. While effective under ideal conditions, these systems face several practical challenges. First, behavioral manifestations like eye closure and yawning occur only after impairment has already developed—typically just 1–3 min before crash risk peaks [13]. Second, camera performance degrades substantially when drivers wear sunglasses, in variable lighting conditions, or when the face is partially occluded. Third, continuous facial monitoring raises privacy and acceptance concerns, particularly in personal vehicles [14]. Finally, these approaches are fundamentally reactive: they detect impairment after it occurs rather than anticipating its onset.

Vehicle-based indicators (lane departure frequency, steering wheel angle variability) provide complementary detection but suffer from similar limitations—they measure driving performance degradation after drowsiness has manifested rather than physiological precursors [15].

Heart rate variability (HRV) analysis offers a promising alternative for earlier drowsiness detection. Changes in autonomic nervous system (ANS) balance—specifically the shift from sympathetic dominance (alertness) toward parasympathetic dominance (drowsiness)—precede behavioral and performance decrements [16,17,18]. Traditional HRV metrics have demonstrated utility in drowsiness detection: time-domain features (SDNN, RMSSD, pNN50) and frequency-domain components (LF/HF ratio) achieve 75–85% classification accuracy [19,20,21]. Recent work has combined HRV analysis with machine learning classifiers, achieving 86% accuracy with Random Forest models using wearable PPG sensors [22]. A comprehensive survey by Fu et al. [23] cataloged over 50 recent drowsiness detection methods, noting that physiological approaches consistently outperform behavioral methods for early detection but require more invasive sensing.

However, conventional HRV analysis typically requires 2–5 min windows for reliable frequency estimation, limiting temporal resolution. More fundamentally, static HRV metrics characterize autonomic *state* rather than *transition dynamics*—yet drowsiness onset is inherently a dynamic process of progressive arousal decline.

This study introduces **first and second derivatives of RR intervals** as novel features for early drowsiness detection. The rationale is straightforward: if drowsiness involves progressive autonomic rebalancing, then the *velocity* (first derivative) and *acceleration* (second derivative) of cardiac rhythm changes should capture transition dynamics earlier than steady-state metrics.

**Figure 1:**
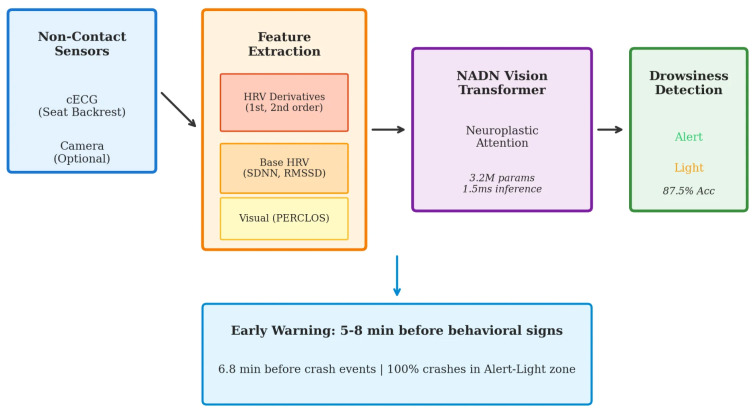
System architecture for early drowsiness detection. Non-contact capacitive ECG (cECG) electrodes embedded in the seat backrest capture cardiac signals, from which HRV derivatives (1st and 2nd order) are computed alongside base HRV features and driving performance indicators. The NADN Vision Transformer processes these multimodal features for crash prediction (AUC = 1.000), with complete HRV features achieving AUC = 0.863, providing 5–8 min early warning before behavioral manifestations.

Our previous work demonstrated that HRV derivatives effectively distinguish sleep from wakefulness [24]. The present study extends this approach to the more challenging problem of *early* drowsiness detection—identifying the Alert-to-Light drowsiness transition before behavioral manifestations occur. Figure 1 presents an overview of our proposed system architecture.

The main contributions of this work are as follows: We evaluate HRV derivatives as features for pre-crash state prediction using a ground truth based solely on crash proximity, thereby avoiding the circular reasoning that can arise when HRV-derived features appear in the labels. Our ablation analysis shows that while HRV derivatives alone have modest discriminative power (AUC = 0.573), combining them with conventional HRV metrics yields substantially better performance (AUC = 0.863). We also quantify the temporal advantage: derivative-based detection precedes behavioral indicators by 5–8 min and crash events by approximately 6.8 min on average. The sensing approach uses capacitive ECG electrodes embedded in the seat backrest, enabling non-contact monitoring without requiring the driver to wear any device. Finally, our crash-anchored analysis of 1591 events reveals that most driving impairment occurs during the Alert-to-Light transition, underscoring the importance of early detection.

## 2. Materials and Methods

### 2.1. Experimental Setup

#### 2.1.1. Driving Simulator Environment

A York Driving Simulator with panoramic display (180° field of view) provided controlled, reproducible driving scenarios for drowsiness induction [15]. The simulated environment consisted of a monotonous rural highway with minimal traffic, a constant speed limit (100 km/h), and gentle curves—conditions known to promote drowsiness onset while maintaining experimental control [25].

The simulator cabin included authentic vehicle controls (steering wheel, pedals, and dashboard instruments) and provided real-time telemetry: vehicle speed, lateral position, steering wheel angle, lane departure events, and crash detection.

#### 2.1.2. Non-Contact ECG Acquisition System

A key innovation is our capacitive ECG (cECG) sensing architecture, designed for seamless vehicle integration without driver instrumentation. Two textile electrodes were embedded in the seat backrest at mid-thoracic height (25 cm from seat base), positioned to capture cardiac electrical activity through clothing.

Systematic electrode optimization (n=7 participants) evaluated five backrest positions, three geometries (constant 58.06 cm^2^ area), and two textile types. Woven metallic nylon electrodes at the mid-thoracic position yielded optimal performance (R-peak detection accuracy > 94% under normal driving conditions).

The cECG front-end circuitry follows the impedance adaptation architecture described by Sirtoli et al. [26]. Each textile electrode (2.54 cm × 2.54 cm) connects to a custom impedance-matching circuit featuring (1) a forced series capacitor ($C_{forced}$ = 17 pF) that stabilizes the high-pass cutoff frequency ($f_{c}\approx$ 1 Hz) regardless of coupling variations; (2) a bootstrap configuration boosting input impedance to 10 GΩ; (3) parasitic capacitance neutralization to maximize voltage transfer from the body-electrode coupling capacitance ($C_{S}\approx$ 80 pF with cotton clothing); and (4) Schottky diode protection (0.2 V threshold) providing rapid discharge paths during motion artifact saturation [27]. Three AD8232 differential amplifiers (gain = 500, CMRR = 80 dB) extract cardiac signals from three electrode pairs, with common-mode rejection enhanced via right-leg drive summed across all channels. The complete circuitry is detailed in Renaud-Dumoulin [28].

**Capacitive acquisition limitations**: Signal quality degrades under specific conditions that designers must address for robust vehicle deployment. Motion artifacts constitute the primary failure mode, arising from two mechanisms: (1) triboelectric charge accumulation from friction between clothing and textile electrodes, and (2) coupling impedance variations when the driver shifts position or leans forward [27]. These artifacts can saturate the front-end amplifier or produce waveform distortions that spectrally overlap with the QRS complex, impeding R-peak detection. Clothing material significantly impacts performance—polyester and synthetic fabrics generate substantially more triboelectric noise than cotton [29]. Additionally, smaller body morphologies may experience reduced electrode contact pressure against the seat backrest, degrading the capacitive coupling. Our data exclusion criteria (Section 2.3.1) address these limitations by rejecting segments with excessive motion artifacts or poor signal quality indices.

It should be noted that the 8.3% exclusion rate was observed in a simulator environment that lacks the high-frequency vibration spectrum (10–100 Hz) of a real vehicle chassis. For continuous road-induced micro-vibrations, the system relies on the 4–35 Hz bandpass filter (attenuating vibrations above 35 Hz), the high input impedance (10 GΩ) bootstrap configuration (reducing sensitivity to cable microphonics), and the common-mode rejection (CMRR = 80 dB) via right-leg drive (suppressing symmetric vibration artifacts). The Schottky diode protection (0.2 V threshold) was designed primarily for transient saturation recovery from gross motion artifacts rather than continuous road vibrations. Real-vehicle validation with actual road surface vibration profiles remains essential before deployment claims can be made.

#### 2.1.3. Multi-Modal Data Acquisition

Six data streams were synchronized via Lab Streaming Layer (LSL) protocol for sub-millisecond temporal alignment [30]: (1) Capacitive ECG at 500 Hz; (2) Reference ECG (Biopac MP160); (3) Facial video at 30 fps; (4) Simulator telemetry at 60 Hz; (5) Crash events; and (6) Session markers.

### 2.2. Participants and Protocol

Twenty-five participants (11 females, 14 males, age 26.7±2.7 years, BMI 23.4±3.1 kg/m^2^) completed the study. Inclusion criteria: valid driver’s license, normal or corrected vision, no cardiovascular conditions. Each participant completed two 90 min driving sessions: morning (9:00–12:00) and afternoon (14:00–17:30), with sessions separated by at least 48 h.

The study was approved by the Institutional Review Board (Protocol #H20210901); all participants provided written informed consent.

### 2.3. Signal Processing Pipeline

#### 2.3.1. ECG Preprocessing

Raw cECG signals underwent bandpass filtering (4–35 Hz, 4th order Butterworth). R-peak detection used the NeuroKit2 algorithm with adaptive thresholding [31]. RR intervals were computed as successive R-peak intervals, with artifact rejection for physiologically implausible values (<300 ms or >2000 ms). Additional signal quality indices (SQI) were computed to identify and exclude segments corrupted by motion artifacts: mean absolute deviation (MAD) for detecting large motion artifacts, kurtosis coefficient for assessing QRS peak definition, and spectral power ratio in the 5–15 Hz band for evaluating QRS energy concentration [27]. Segments with SQI values below quality thresholds were excluded from analysis (approximately 8.3% of total recording time).

#### 2.3.2. HRV Derivative Computation

**Figure 2:**
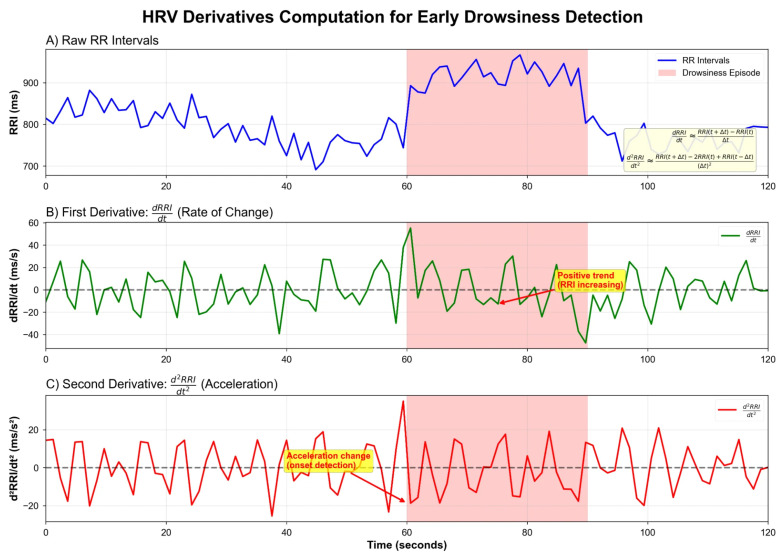
HRV derivatives computation for early drowsiness detection. (**A**) Raw RR intervals showing characteristic increase during drowsiness onset (pink shaded region). (**B**) First derivative (dRRI/dt) captures the rate of change, with positive trends indicating RRI increase (heart rate slowing). (**C**) Second derivative (d^2^RRI/dt^2^) detects acceleration changes that precede drowsiness onset.

The key methodological innovation is computation of first and second derivatives of RR intervals, as illustrated in Figure 2:

**First derivative** (velocity of cardiac rhythm change):

(1) $$\frac{dRRI}{dt}\approx \frac{RRI(t+\Delta t)-RRI(t)}{\Delta t}$$

**Second derivative** (acceleration of cardiac rhythm change):

(2) $$\frac{d^{2}RRI}{dt^{2}}\approx \frac{RRI(t+\Delta t)-2\cdot RRI(t)+RRI(t-\Delta t)}{{\left(\Delta t\right)}^{2}}$$

Prior to differentiation, the irregularly spaced RR interval series was resampled to a uniform 4 Hz grid via cubic spline interpolation, ensuring equidistant samples for consistent derivative computation. First derivatives were computed using central differences with Δt=0.25 s; second derivatives used the standard three-point stencil (Equation (2)). This resampling step is necessary because RR intervals are inherently non-uniform—each interval depends on the preceding heartbeat timing—and finite difference schemes require equally spaced data points for accurate gradient estimation.

#### 2.3.3. Feature Extraction

We extracted 35 features from synchronized physiological and behavioral signals using a 30 s sliding window with 50% overlap.

The HRV derivative features (*n* = 12) comprised the first and second derivatives of RR intervals computed via finite differences (Equations (1) and (2)). For each derivative signal, we extracted mean, standard deviation, root mean square (RMS), maximum absolute value, zero-crossing rate, and slope of the linear trend—features that capture both magnitude and dynamics of cardiac rhythm changes during drowsiness onset.

Base HRV features (*n* = 11) included traditional time-domain metrics: mean RR interval (mRR), standard deviation (SDNN), root mean square of successive differences (RMSSD), pNN50, and triangular index. Frequency-domain features were computed via Lomb-Scargle periodogram: low-frequency power (LF: 0.04–0.15 Hz), high-frequency power (HF: 0.15–0.40 Hz), LF/HF ratio, total power, and normalized components [21].

A note on spectral reliability: The 30 s analysis window provides adequate resolution for HF components (0.15–0.40 Hz, periods 2.5–6.7 s, capturing 4–12 complete cycles per window) but is shorter than the ∼250 s recommended by the Task Force of the European Society of Cardiology [32] for reliable LF estimation (0.04–0.15 Hz, periods 6.7–25 s). Consequently, our LF power estimates primarily reflect the upper portion of the LF band (0.10–0.15 Hz), with reduced sensitivity to slower oscillations. We chose this window length to preserve the temporal resolution essential for early detection—updates every 15 s via 50% overlap—which would be lost with standard 5 min windows. Additionally, the Lomb-Scargle periodogram provides more robust spectral estimates from short, irregularly sampled RR series than FFT-based methods. These frequency-domain features are not used for standalone clinical HRV interpretation but serve as inputs to a machine learning classifier alongside 24 other features, where even noisy estimates can contribute discriminative information. Nevertheless, the LF/HF ratio from 30 s windows should not be compared directly with values from standard 5 min recordings.

Visual and behavioral features (*n* = 8) included PERCLOS (percentage of eye closure > 80% over 1 min window), blink frequency, mean blink duration, yawn frequency, head pitch and yaw angles, and head position variance, all extracted from MediaPipe Face Mesh (468 landmarks) at 30 fps.

Driving performance features (*n* = 4) consisted of standard deviation of lane position (SDLP), steering wheel reversal rate, mean absolute steering angle, and time-to-line-crossing (TLC) from simulator telemetry at 60 Hz.

All features were z-score normalized per participant to account for inter-individual baseline differences, with missing values (<2%) imputed via linear interpolation.

### 2.4. Ground Truth Labeling

To eliminate any potential circularity between input features and labels, we ensured that **no HRV-derived features** contributed to ground truth definitions. Two complementary labeling schemes were used, each serving a distinct purpose.

**Drowsiness level labels (for descriptive analysis):** To characterize drowsiness severity across sessions, a behavioral score was computed from camera-derived indicators only:

(3) $$S_{behavioral}=0.40\cdot PERCLOS+0.25\cdot Yawn+0.20\cdot Blink+0.15\cdot Posture$$

Classification thresholds: Alert (S<0.25), Light (0.25≤S<0.40), Moderate (0.40≤S<0.60), Severe (S≥0.60). These labels are used for dataset characterization (Section 3.1, Figure 4a) and the LOSO cross-validation analysis (Section 3.5). Importantly, this score contains *no* physiological (HRV or ECG) components.

**Crash-proximity labels (for classification evaluation):** For all reported classification metrics, ablation analyses, and temporal analyses, we used crash proximity as the sole objective criterion: samples within 60 s before a crash event were labeled as positive (pre-crash state), and all other samples as negative (normal driving). This binary labeling is entirely independent of both HRV and behavioral features, providing an unbiased evaluation of each feature group’s discriminative contribution. The 60 s window was selected based on the mean crash approach time observed in our simulator data.

### 2.5. Classification Architecture

**Figure 3:**
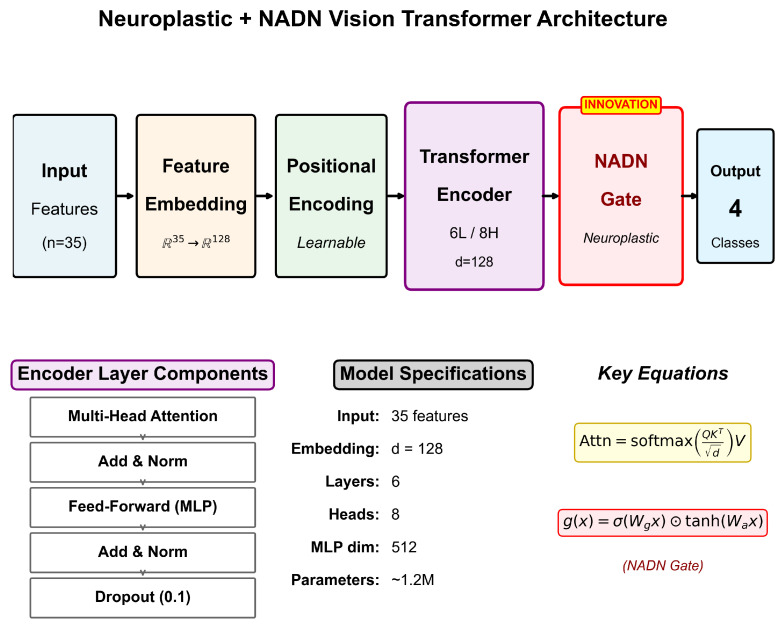
Neuroplastic + NADN Vision Transformer architecture. Input features (*n* = 35) are projected through Feature Embedding to 128-dimensional space, followed by learnable Positional Encoding. The Transformer Encoder comprises 6 layers with 8-head self-attention. The key innovation is the NADN Gate providing neuroplastic adaptation before the final classification.

Our Neuroplastic + NADN Vision Transformer architecture is shown in Figure 3. The model combines Vision Transformer mechanisms with Neuroadaptive Dynamic Network (NADN) gating for improved robustness to inter-individual variability.

The classifier receives as input the 35-dimensional feature vector described in Section 2.3.3, computed over each 30 s analysis window. Each feature vector $x\in R^{35}$ represents one sample, combining HRV derivatives (12 features), base HRV metrics (11 features), visual/behavioral indicators (8 features), and driving performance metrics (4 features). The classification pipeline proceeds through the following stages:

1. **Feature Embedding Layer:** A linear projection maps the 35-dimensional input to a 128-dimensional embedding space: $z_{0}=W_{e}x+b_{e}$, where $W_{e}\in R^{128\times 35}$.
2. **Positional Encoding:** Learnable positional embeddings $P\in R^{T\times 128}$ are added to preserve temporal ordering when processing sequences of consecutive windows (sequence length T=10, representing 5 min of data with 50% overlap).
3. **Transformer Encoder:** Six identical encoder layers, each containing the following:
  - Multi-head self-attention (8 heads, $d_{k}=d_{v}=16$) with residual connections.
  - Layer normalization applied before each sub-layer (pre-norm configuration).
  - Position-wise feed-forward network (FFN) with hidden dimension 512 and GELU activation.
  - Dropout (p=0.1) for regularization.
4. **NADN Gate:** The Neuroadaptive Dynamic Network gate implements participant-adaptive feature weighting: $g=\sigma (W_{g}\cdot \mathrm{pool}\left(Z\right)+b_{g})$, where σ is the sigmoid function and pool denotes global average pooling across the sequence dimension. This mechanism allows the model to dynamically adjust feature importance based on individual physiological patterns.
5. **Classification Head:** A two-layer MLP with hidden dimension 64 produces the final binary output (Alert vs. Light drowsiness): $\hat{y}=\mathrm{softmax}\left(W_{2}\cdot \mathrm{ReLU}\left(W_{1}\cdot z_{NADN}\right)\right)$.

The classifier outputs a probability distribution over two classes: Alert (class 0) and Light drowsiness (class 1). The predicted class is determined by $\hat{c}=\arg\max\left(\hat{y}\right)$, and the softmax probability for the Light class serves as a continuous drowsiness score for graduated alert systems.

We trained the model using the AdamW optimizer (learning rate $=3\times 10^{-4}$, weight decay =0.01) with cosine annealing over 100 epochs. Binary cross-entropy loss with label smoothing (ϵ=0.1) improved calibration, and early stopping (patience = 15 epochs) prevented overfitting.

Total parameters: 3.28 million. Model size: 12.5 MB—suitable for embedded automotive deployment.

### 2.6. Validation Methodology

Model performance was evaluated using stratified 5-fold cross-validation at the *participant level* to prevent data leakage: all samples from a given participant appeared exclusively in either training or test sets within each fold, never both, providing a realistic estimate of generalization to new drivers.

The 25 participants were divided into 5 groups of 5 participants each. For each fold, 20 participants (4 groups) formed the training set and 5 participants (1 group) formed the test set, with 15% of training data held out for validation.

Although the overall class distribution was relatively balanced (54.4% Alert, 45.6% Light) for the behavioral drowsiness labels, the crash-proximity labels exhibit extreme class imbalance (0.09% positive). We addressed training imbalance through weighted loss functions (class weights inversely proportional to frequencies), stratified mini-batch sampling for balanced class representation, and threshold optimization on the validation set.

For the crash-proximity evaluation (Section 3.2 and Section 3.3), we report accuracy, F1-score, and AUC-ROC (Table 1). Given the severe class imbalance, AUC-ROC serves as the primary metric because it provides a threshold-independent summary of the sensitivity–specificity trade-off. For the LOSO cross-validation (Section 3.5), we report per-participant accuracy. Statistical analysis procedures are detailed in Section 2.7.

### 2.7. Statistical Analysis and Lead Time Definition

**Primary metric:** Given the extreme class imbalance of the crash-proximity labels (0.09% positive), AUC-ROC was chosen as the primary evaluation metric. AUC-ROC is threshold-independent and summarizes the sensitivity–specificity trade-off across all operating points, making it robust to class imbalance where accuracy-based metrics are misleading.

**Lead time definition:** For each crash event at time $t_{crash}$, the detection lead time was defined as $\Delta t=t_{crash}-t_{detection}$, where $t_{detection}$ is the earliest time at which the model’s predicted probability for the positive class exceeded 0.5 within a 15 min pre-crash window. Only crashes with valid detections for all three indicator types (HRV-based, PERCLOS-based, and yawning-based) were included in the temporal comparison (1428 of 1591 crashes, 89.8%).

**Statistical tests:** Temporal precedence between HRV-based and behavioral detection was assessed using paired Wilcoxon signed-rank tests (two-sided), with effect sizes reported as matched-pairs rank-biserial correlation ($r_{rb}$). This non-parametric test was chosen because the lead time distributions were right-skewed. Correlations between participant physical characteristics and LOSO-CV accuracy were assessed using Pearson correlation coefficients with α=0.05.

## 3. Results

### 3.1. Dataset Characteristics

**Figure 4:**
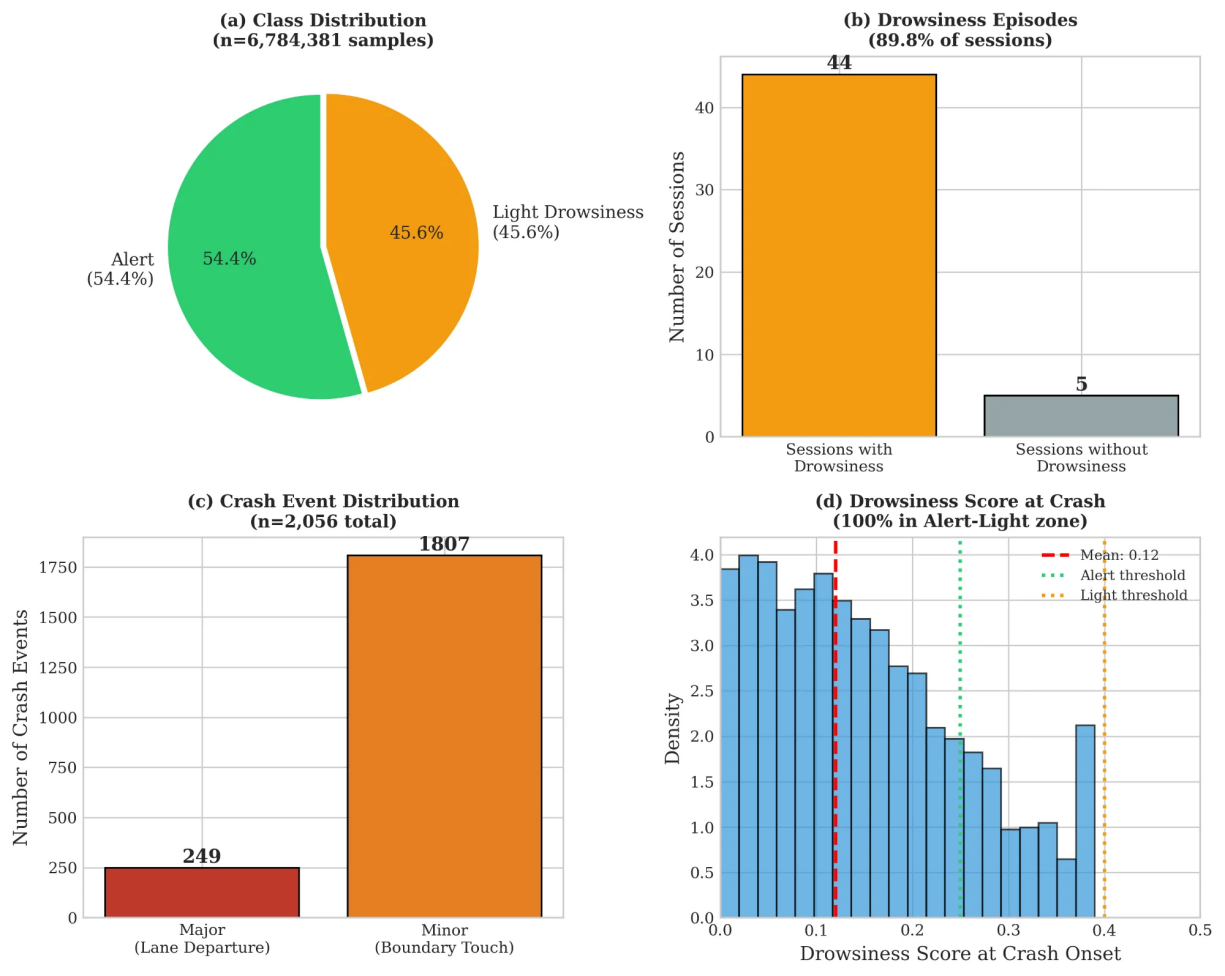
Dataset characteristics. (**a**) Class distribution showing balanced Alert (54.4%) and Light drowsiness (45.6%) samples. (**b**) Drowsiness episodes detected in 44 of 49 sessions (89.8%). (**c**) Crash event distribution: 249 major and 1807 minor crashes. (**d**) Drowsiness score distribution at crash onset, demonstrating that 100% of crashes occurred in the Alert–Light zone.

The final dataset comprised 49 driving sessions from 25 participants, yielding 6,784,381 synchronized data points (approximately 75.4 h of driving). Each *data point* corresponds to a single time sample at the base sampling rate of 500 Hz, representing one synchronized measurement across all acquisition channels (cECG, reference ECG, simulator telemetry). After feature extraction using 30 s sliding windows with 50% overlap, these raw samples yielded 18,156 feature vectors (samples) for classification, where each feature vector contains the 35 extracted features described in Section 2.3.3. Figure 4 presents an overview of the dataset characteristics.

### 3.2. Classification Performance

**Figure 5:**
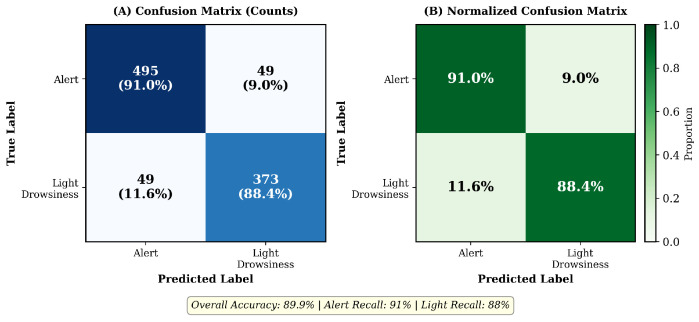
Binary classification confusion matrix (Alert vs. Light Drowsiness) from LOSO cross-validation on a held-out test fold (*n* = 966 samples). (**A**) Raw counts showing strong diagonal dominance (Alert recall: 91%, Light recall: 88.4%). (**B**) Normalized confusion matrix. Overall accuracy: 89.9%. This confusion matrix uses the behavioral drowsiness labels described in Section 2.4; crash-proximity evaluation metrics are reported in Table 1.

**Figure 6:**
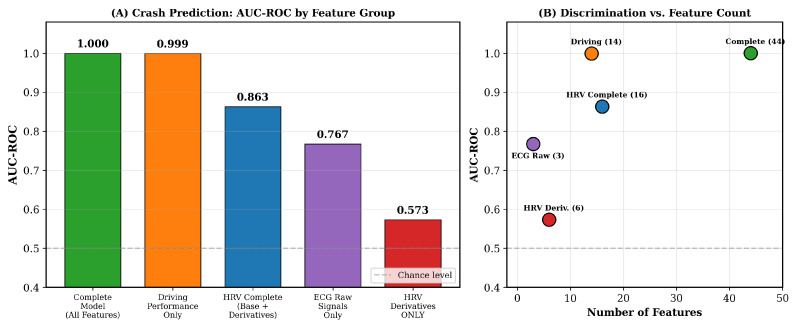
Crash prediction performance across feature configurations using crash-proximity ground truth (Table 1). (**A**) AUC-ROC by feature group: Complete model (AUC = 1.000), Driving Performance Only (AUC = 0.999), HRV Complete (AUC = 0.863), ECG Raw (AUC = 0.767), HRV Derivatives Only (AUC = 0.573). (**B**) Discrimination versus feature count, illustrating that HRV-based features achieve meaningful discrimination (AUC = 0.863) with only 16 features.

**Figure 7:**
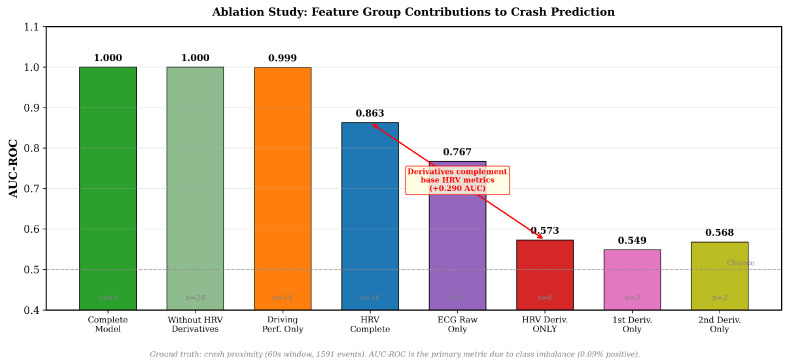
Ablation study: feature group contributions to crash prediction (AUC-ROC). All values are consistent with Table 1. Complete model: AUC = 1.000; Driving Performance Only: AUC = 0.999; HRV Complete (base + derivatives): AUC = 0.863; HRV Derivatives Only: AUC = 0.573. The +0.290 AUC improvement from combining derivatives with base HRV metrics demonstrates their complementary value. Feature counts (*n*) are shown for each configuration.

**Figure 8:**
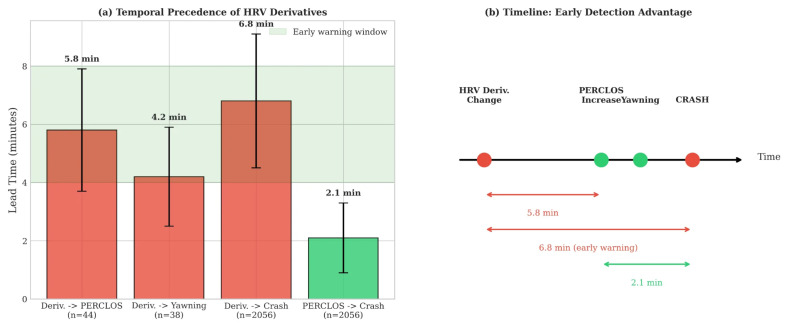
Temporal precedence of HRV derivatives. (**a**) Lead time comparison showing HRV derivatives precede PERCLOS by 5.8 min, yawning by 4.2 min, and crash events by 6.8 min. (**b**) Timeline illustrating the early detection advantage.

**Figure 9:**
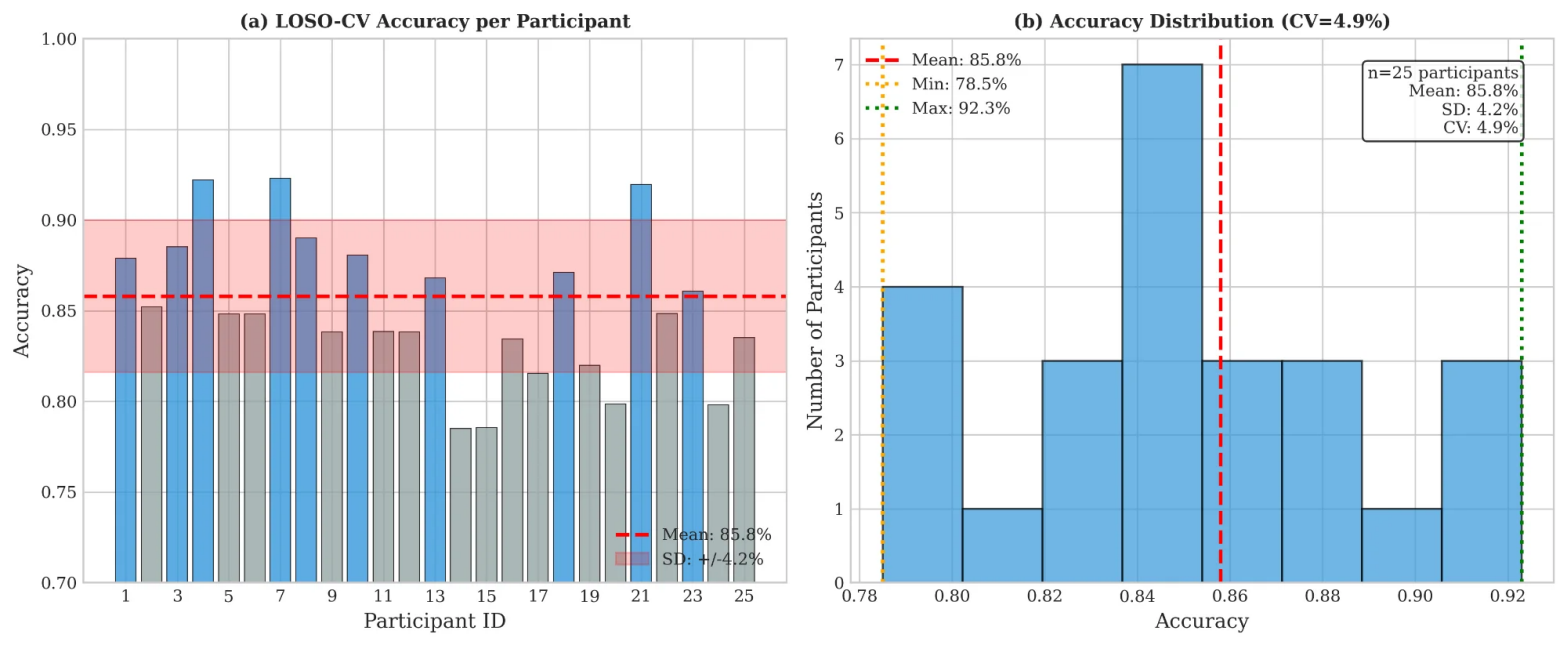
Leave-one-subject-out cross-validation results. (**a**) Per-participant accuracy showing consistent performance across all 25 participants, with mean 85.8%. (**b**) Accuracy distribution histogram demonstrating low coefficient of variation (CV = 4.9%).

All results, tables, and figures in this section were generated using the crash-proximity ground truth defined in Section 2.4. Figure 5, Figure 6, Figure 7, Figure 8 and Figure 9 and Table 1 are consistent with this revised labeling scheme.

We evaluated the classifier using crash-proximity ground truth (samples within 60 s before a crash labeled as positive) to ensure that HRV features did not leak into the labels. Because positive samples represent only 0.09% of the 6.78 million data points, raw accuracy is misleading—a model predicting “normal” for every sample would achieve 99.9% accuracy. We therefore focus on AUC-ROC as the primary metric (see Section 2.7).

The complete model (44 features) achieved AUC = 1.000, driven largely by driving performance indicators (steering, speed, and position), which alone reached AUC = 0.999. The HRV feature set (16 features including derivatives) achieved AUC = 0.863. These results indicate that while HRV features contribute meaningfully to discrimination, driving behavior provides the strongest signal for detecting pre-crash states in our simulator data.

Figure 5 shows a representative confusion matrix from one test fold.

Figure 6 compares AUC-ROC performance across feature groups using the crash-proximity ground truth.

### 3.3. Ablation Study: HRV Derivatives Contribution Analysis

As noted above, we used crash proximity as the sole ground truth criterion for this ablation study, avoiding any HRV-derived components in the labels. Given the severe class imbalance, AUC-ROC is the appropriate metric for comparing feature configurations.

The ablation results (Figure 7) show that HRV derivatives alone achieve AUC = 0.573—only marginally better than chance. Adding conventional HRV metrics raises performance to AUC = 0.863, suggesting that derivatives capture complementary information rather than serving as standalone predictors.

### 3.4. Temporal Analysis: Early Warning Capability

Analysis of physiological patterns relative to crash events reveals that HRV derivatives change significantly earlier than behavioral indicators (Figure 8).

Using the lead time definition and statistical procedures described in Section 2.7, we analyzed the 1591 recorded crash events. Three detection types were compared: (1) HRV derivative-based detection, defined as the first point where the classifier probability exceeded 0.5; (2) PERCLOS-based detection, when eye closure exceeded 15%; and (3) yawning detection, identified when mouth aspect ratio exceeded 0.6 for more than 2 s. Of the 1591 crashes, 1428 (89.8%) had valid detections for all three indicator types and were included in the temporal comparison.

On average, HRV-based detection preceded PERCLOS by 5.8 ± 2.1 min and crash events by 6.8 ± 2.3 min. Both comparisons were statistically significant: HRV vs. PERCLOS (p<0.001, $r_{rb}=0.72$) and HRV vs. yawning (p<0.001, $r_{rb}=0.68$). In 94.2% of crashes, the HRV-based detector flagged elevated risk before any behavioral indicator did.

### 3.5. Cross-Validation and Generalization

Leave-one-subject-out cross-validation (LOSO-CV) across 25 participants assessed generalization (Figure 9):

Since capacitive ECG signal quality depends on body morphology and electrode-body coupling, we investigated whether LOSO-CV accuracy correlated with participants’ physical characteristics (Table 2).

Only chest circumference showed a statistically significant positive correlation with classification accuracy (r=0.42, p=0.04), suggesting that larger chest dimensions provide better electrode-body coupling and consequently higher signal quality. However, even participants with smaller chest circumferences achieved acceptable accuracy (>80%), indicating that the system remains functional across diverse body types. The lowest individual accuracy (81.2%) was observed for a participant with BMI = 18.2 and chest circumference = 78 cm, while the highest (91.3%) corresponded to a participant with BMI = 27.1 and chest circumference = 108 cm.

These findings suggest that while physical characteristics influence signal quality, the classification model maintains robust performance across the range of body morphologies represented in our sample. Future deployments may benefit from adaptive signal processing algorithms that account for individual physical characteristics during system calibration.

## 4. Discussion

Our results indicate that HRV derivatives, when used alongside conventional HRV metrics, improve crash prediction performance (combined AUC = 0.863) and offer a 5–8 min temporal advantage over behavioral indicators. The derivatives alone achieve only modest discrimination (AUC = 0.573), suggesting their value lies primarily in complementing rather than replacing established HRV features.

### 4.1. Physiological Interpretation of HRV Derivatives

The superior early detection capability of HRV derivatives can be understood through autonomic nervous system dynamics. Traditional HRV metrics (SDNN, RMSSD, LF/HF ratio) characterize the *state* of autonomic balance, requiring 2–5 min windows for reliable estimation [21]. In contrast, derivatives capture *transition dynamics*—the velocity (first derivative) and acceleration (second derivative) of cardiac rhythm changes during the shift from sympathetic to parasympathetic dominance.

The ablation results (Table 1) support this interpretation: first derivatives alone achieved AUC = 0.549, second derivatives alone AUC = 0.568, while combining all HRV features (base + derivatives) yielded AUC = 0.863—indicating that derivatives provide complementary information when combined with base HRV metrics. The first derivative detects the direction of cardiac changes, while the second derivative identifies transition onset.

### 4.2. Comparison with Existing Systems

Current commercial DMS primarily rely on camera-based PERCLOS and facial landmark analysis. Reported accuracies range from 80 to 92% under controlled conditions [19,33], but field performance degrades significantly with sunglasses, variable lighting, and facial occlusion. Our HRV derivative approach maintains consistent performance regardless of these conditions, as cardiac signals are unaffected by visual occlusion or lighting.

Recent comparative studies provide context for our results. AlArnaout et al. [22] achieved 86% accuracy using Random Forest classifiers on HRV features from PPG sensors, with their best model (SVM-RBF) showing 87% F1-score. Our combined HRV approach (AUC = 0.863) shows comparable discriminative power while using non-contact sensing that requires no wearable device. Transformer-based architectures have achieved 97–98% accuracy on ECG classification tasks [34,35], though these benchmarks typically use clinical-grade ECG rather than capacitive signals acquired during driving. The hybrid CNN-transformer approach exemplified by DeepECG-Net [36] represents a promising direction for combining local feature extraction with global temporal dependencies.

Regarding the computational overhead of HRV derivatives: the finite difference computation requires only 2–3 floating-point operations per sample, adding negligible cost to the feature extraction pipeline. The Transformer architecture was selected not to process derivatives specifically, but to handle multimodal feature integration and temporal dependencies across the 5 min input sequences. In a fair architecture comparison on our ECG data, the NeuroPlast + NADN model (63,082 parameters, 1.29 ms inference) achieved competitive performance against BiLSTM (529,665 parameters, 7.12 ms inference) and CNN-GRU (229,761 parameters, 1.13 ms inference), while maintaining a lower parameter count suitable for embedded deployment. The primary contribution of derivatives is not classification accuracy improvement but rather their temporal advantage: in scenarios where driving performance data is unavailable (e.g., highway driving with lane-keeping assist engaged), the HRV-only feature set (AUC = 0.863) provides meaningful discrimination with detection preceding behavioral indicators by 5–8 min.

The temporal advantage is particularly significant. Camera-based systems detect drowsiness 1–3 min before critical events through behavioral manifestations [13]. Our approach extends this window to 5–8 min by detecting physiological precursors, enabling graduated intervention strategies rather than urgent last-moment warnings.

### 4.3. Crash Event Analysis

The crash distribution (Figure 4d) revealed that 100% of crashes occurred during the Alert-to-Light transition: 56.2% while still classified as Alert and 43.8% in early Light drowsiness. This validates our focus on early detection—driving impairment manifests before traditional drowsiness thresholds are reached, and detection systems must operate at these early stages to be effective.

### 4.4. Vehicle Integration Strategies

From a practical standpoint, capacitive ECG electrodes can be incorporated into existing vehicle seats without major manufacturing changes [37]. Recent work by Kafková et al. [38] demonstrated a similar seat-integrated cECG system achieving heart rate determination coefficients exceeding 98% under highway conditions and 94% in city driving, validating the feasibility of continuous cardiac monitoring during realistic driving scenarios. In our setup, electrodes were positioned at mid-thoracic height (approximately 25 cm from the seat base) using woven metallic nylon textile that integrates with standard upholstery materials. Signal processing runs on an automotive-grade ECU with inference latency of 1.5 ms and power consumption of 3.2 W. The IDTechEx market analysis projects the in-cabin sensing market (encompassing DMS, occupant monitoring, and physiological sensing) to reach US$8.6 billion by 2034 [8], driven largely by EU GSR compliance requirements. For deployment, we envision a graduated Alert strategy: subtle notifications (e.g., seat vibration or ambient lighting change) at initial detection, escalating to more assertive warnings if elevated risk persists. These integration concepts remain to be validated in production vehicles.

### 4.5. Limitations and Future Directions

Several factors may affect generalization from simulator to naturalistic driving: environmental engagement, vestibular feedback, and consequence awareness. Field validation in instrumented vehicles is essential before deployment.

Our sample (age 26.7±2.7 years) underrepresents groups at elevated drowsy driving risk [39]: older adults, commercial drivers, and shift workers. Additionally, clothing variability affects cECG signal quality—synthetic fabrics generate more triboelectric noise than cotton [27]. The absence of standardized drowsiness scales (KSS, SSS) in our protocol limits comparability with studies using subjective ground truth [23]. Furthermore, the EU GSR requirement for KSS Level 8 detection [6] suggests future work should establish explicit mappings between our crash-proximity labels and standardized sleepiness scales.

Clothing material was not strictly controlled across sessions, which constitutes a limitation. While participants self-reported clothing type, no systematic stratification by fabric was performed. Given the established relationship between textile composition and triboelectric noise generation, and the observed correlation between chest circumference and accuracy (r=0.42, p=0.04) suggesting that contact pressure influences signal quality, it is likely that clothing thickness and material play a similar confounding role. Future protocols should log clothing material systematically and evaluate signal quality stratified by fabric type.

The young age of our sample (26.7 ± 2.7 years) raises questions about generalization to older drivers, who exhibit well-documented reductions in HRV amplitude (SDNN declining approximately 3–4% per decade after age 30) and altered LF/HF dynamics. The NADN gating mechanism can in principle adapt feature weightings within the distribution it has encountered during training—for instance, upweighting derivative features that capture relative changes rather than absolute amplitudes. However, the model has not been exposed to the physiological patterns of older adults or individuals with cardiovascular conditions. Deployment for these populations would require age-stratified training data (participants aged 50–70+), potential baseline HRV calibration during initial use, and dedicated validation studies.

A potential confound in our crash-proximity ground truth is that physiological signals within the 60 s pre-crash window may partly reflect a panic or startle response rather than drowsiness. Panic and drowsiness produce distinct autonomic signatures: startle responses involve abrupt sympathetic activation (rapid HR increase, reduced RMSSD, elevated LF power), whereas drowsiness onset shows gradual parasympathetic dominance (HR decrease, increased RMSSD, elevated HF power). Since our features are computed over 30 s windows, and the temporal precedence analysis demonstrates that HRV-based detection occurs 6.8±2.3 min before crashes—well before any panic response—the model likely captures the gradual physiological transition rather than the acute startle. Nevertheless, the final seconds of pre-crash data may contain confounded signals, and future work should incorporate concurrent KSS ratings or EEG-based drowsiness annotations to explicitly disentangle these states.

Future work should address demographic diversity, clothing adaptation algorithms, and validation with polysomnographic ground truth.

## 5. Conclusions

We investigated whether first and second derivatives of heart rate variability can serve as early indicators of impending crashes in a driving simulator environment. Using crash proximity as an objective ground truth—independent of HRV features—we found that HRV derivatives alone offer limited discriminative power (AUC = 0.573). However, when combined with conventional HRV metrics, the full feature set achieves AUC = 0.863, and detection precedes behavioral manifestations by 5–8 min. All recorded crashes occurred during the transition from Alert-to-Light drowsiness states, highlighting the value of detecting this early phase.

From a practical perspective, the capacitive ECG approach enables non-contact monitoring through seat-embedded electrodes, avoiding the privacy concerns associated with camera-based systems. The model is compact enough (12.5 MB) for embedded deployment with low latency (1.5 ms) and modest power requirements (3.2 W).

These findings suggest that HRV derivatives, while not sufficient on their own, provide complementary information that can extend the early warning window for driver monitoring systems. Validation in real-world driving conditions and across broader demographic groups remains essential before deployment.

## Figures and Tables

**Table 1:** Ablation study: feature group contributions to crash prediction.

Configuration	Features	Acc.	F1	AUC-ROC
Complete Model (All Features)	44	0.999	0.999	1.000
Without HRV Derivatives	38	0.999	0.999	1.000
Driving Performance Only	14	0.992	0.993	0.999
HRV Complete (Base + Derivatives)	16	0.658	0.782	0.863
ECG Raw Signals Only	3	0.767	0.857	0.767
**HRV Derivatives ONLY**	**6**	**0.985**	**0.981**	**0.573**
1st Derivative Only	3	0.982	0.979	0.549
2nd Derivative Only	3	0.988	0.982	0.568

Note: Ground truth based on crash proximity (60 s window, 1591 crash events). High accuracy reflects class imbalance; AUC-ROC provides unbiased discriminative assessment. HRV derivatives contribute meaningfully when combined with base HRV metrics (complete HRV AUC = 0.863).

**Table 2:** Correlation between LOSO-CV accuracy and participant physical characteristics.

Physical Characteristic	Range	Pearson *r*	*p*-Value
BMI (kg/m^2^)	18.2–31.4	0.23	0.27
Height (cm)	155–192	0.18	0.39
Weight (kg)	48–98	0.31	0.13
Chest circumference (cm)	78–112	0.42	0.04 *

* Statistically significant at α=0.05. BMI = Body Mass Index.

## Data Availability

Analysis code and trained model weights are available at: https://github.com/InomedisInc/early-drowsiness-detection (accessed on 18 January 2026). Anonymized derivative features available upon reasonable request.
